# Subclinical Left Ventricular Dysfunction in Children and Adolescence With Thalassemia Intermedia

**DOI:** 10.3389/fped.2022.774528

**Published:** 2022-06-17

**Authors:** Roya Isa Tafreshi, Mohammad Radgoodarzi, Kadijeh Arjmandi Rafsanjani, Fahimeh Soheilipour

**Affiliations:** ^1^Department of Pediatric Cardiology, Ali Asghar Children’s Hospital, School of Medicine, Iran University of Medical Sciences, Tehran, Iran; ^2^Department of Pediatrics, School of Medicine, Iran University of Medical Sciences, Tehran, Iran; ^3^Department of Pediatric Hematology and Oncology, Ali Asghar Children’s Hospital, School of Medicine, Iran University of Medical Sciences, Tehran, Iran; ^4^Department of Pediatric Endocrinology, Minimally Invasive Surgery Research Center, Aliasghar Hospital, School of Medicine, Iran University of Medical Sciences, Tehran, Iran

**Keywords:** thalassemia intermedia, cardiac complications, left ventricular dysfunction, children, echocardiography

## Abstract

**Background:**

Cardiac complications are important causes of morbidity in patients with thalassemia intermedia (TI). We aimed to assess left ventricular (LV) function, using new tissue Doppler imaging (TDI) indices, in order to diagnose early ventricular impairment in asymptomatic children and adolescence with the TI.

**Materials and Methods:**

We investigated possible differences in echocardiographic systolic and diastolic parameters between a population of 28 asymptomatic patients (mean age, 13.6 ± 5.7 years) and 35 age-matched healthy control members. All of them underwent 2-D, pulsed Doppler, and tissue Doppler echocardiographic studies for the assessment of the LV mass, Trans-mitral velocities, mitral annular systolic and diastolic velocities, myocardial performance index (MPI), and myocardial acceleration during isovolumic contraction (IVA). The cardiac iron load was estimated by magnetic resonance imaging T2*.

**Results:**

Left ventricular hypertrophy (LVH) was found in 13 (46.4%) patients. We found significantly reduced TDI-derived peak systolic myocardial velocity (s′) in patients, whereas no significant difference was identified between the patients and control group members when the IVA was compared. The ratio of peak mitral inflow velocity to annular early diastolic velocity (E/e′) of the mitral valve as an index of the diastolic function was significantly higher in patients (9 ± 1 vs. 6 ± 1, *p* < 0.05). Choosing a TDI-derived MPI > 0.33 as a cutoff point, the global LV dysfunction was detected with a sensitivity of 78% and a specificity of 80%. The patients with LVH significantly exhibited higher values of TDI-MPI and lower values of s′ velocity and IVA when compared against the subjects with normal LV mass.

**Conclusion:**

Subtle LV systolic and diastolic dysfunction develops early in young patients with the TI who have normal cardiac iron concentration. Moreover, LV remodeling as a main cardiac adaptive response plays a principal role in developing myocardial impairment.

## Introduction

Thalassemia disorders are the most common inherited hemoglobinopathies with high prevalence in certain regions of the world. Even though individual patients with thalassemia intermedia (TI) have less severe hemolytic anemia than the thalassemia major, they have a heterogeneous clinical presentation (1–4).

Cardiac involvement is one of the most common age-related causes of morbidity in these patients (1, 5, 6). Despite the absence of transfusion-dependent myocardial iron overload, patients with TI may be at risk of iron-related cardiovascular abnormalities as well (7, 8).

Cardiac involvement has been mainly characterized by pulmonary hypertension followed by right heart failure in middle-aged patients with TI; however, recent reports have revealed subtle left ventricular (LV) impairment and remodeling in young patients who have no clinical sign of cardiac failure (9–12). In the absence of iron overload cardiomyopathy, multiple hemodynamic factors may exert an important pathologic effect on the cardiovascular system (1, 5, 9). Chronic hemolytic anemia, ineffective erythropoiesis, tissue hypoxia, and their compensatory reactions contribute to a high cardiac output state, which is a constant finding in patients with TI. The combination of a high cardiac output state with vascular involvement associated with increased pulmonary and systemic vascular resistances is considered the main basic pathophysiologic mechanism for cardiovascular complications in patients with TI (5, 11, 13, 14). Till present, there has been no effective treatment for overt heart failure and, thus, those strategies for preventing or methods for early diagnosis of myocardial tissue damage may improve long-term prognosis.

Most of the previously published echocardiographic studies revealed conflicting information on the LV function, mainly because of the strong influence of high output states on the echocardiographic indices and the unique pattern of left ventricular geometry (11, 13, 15, 16). In this respect, more reliable and less load-dependent parameters should be utilized to identify early ventricular dysfunction. Previous studies have demonstrated that systolic functions, mainly LVEF, remain generally within the normal limit while subtle myocardial abnormalities can be detected using tissue Doppler imaging (TDI) (17, 18).

Although latent LV abnormalities have been studied in the adult with TI, there is less research on LV function in children and adolescence (9, 11).

Thus, the aim of our research was to evaluate the LV function and also LV remodeling, using new pulsed Doppler (PWD) and tissue Doppler echocardiographic indices in young TI subjects with normal cardiac iron concentration to identify the latent myocardial involvement. Also, we assessed the utility of a new tissue Doppler parameter, the myocardial acceleration during isovolumic contraction (IVA), as a marker of ventricular contractility among the patients (19–21).

To the best of our knowledge, there is no previous report on the utility of these new echocardiographic indices for the diagnosis of subclinical LV dysfunction in children and adolescents with TI.

## Materials and Methods

We enrolled 28 consecutive patients with TI (mean age, 13.6 ± 5.7 years), who were referred for their routine cardiac follow-up. Data from all the patients were retrieved for clinical and hematological evaluation. None of the patients had received a regular transfusion or chelating agents. The inclusion criteria were as follows: (1) age 7–21 years, (2) asymptomatic for heart failure (NYHA functional class of I), (3) normal renal and thyroid function, and (4) the absence of congenital or acquired heart disease. The mean serum ferritin value and mean hemoglobin level have been derived from the mean of the three values obtained over the last 3 years. The cardiac iron load was measured by magnetic resonance imaging T2*(MRI T2*). Cardiac iron overload was described as cardiac T2* < 20 ms. A total of 35 age-matched healthy children were selected as controls from cases referred to our department for investigation of a heart murmur.

The cardiac investigation was performed during the periodic follow-up of the patients. An echocardiographic study was performed within 6 months of cardiac MRI evaluation. It is worth mentioning that the protocol was approved by our institutional review board.

### Echocardiographic Examination

Complete M-mode, two-dimensional, Pulsed Doppler (PWD), and tissue Doppler echocardiographic examinations were performed by a physician who was unaware of the status of the cases.

Measurements were performed as stated in the guidelines of the American Society of Echocardiography (22). All of the pulsed Doppler and tissue Doppler-derived parameters were recorded at a sweep speed of 100 mm/s on three consecutive heartbeats, and the average for each was obtained. Electrocardiographic tracing was recorded simultaneously.

All of the study subjects were in sinus rhythm. All of the echocardiographic findings were compared between the TI cases and control subjects.

#### Assessment of Left Ventricular Diameter and Mass

Chamber diameters and wall thicknesses were measured by M-mode echocardiography. We measured LV mass (LVM) using two-dimensionally directed M-mode echocardiography. The LVM was calculated using the formula as previously described (23). The LV mass index (LVMI) was calculated by dividing the LV mass by height to the 2.7 power. The LVH was defined as the LVMI of greater than 51 g/h^2.7^ (22, 24). The LV diameters were also indexed to the body surface area.

#### Assessment of Left Ventricular Systolic and Diastolic Function

The LV ejection fraction (LVEF), stroke volume, and cardiac index were obtained using the biplane Simpson’s methods (22). Reduced LV contractility was defined as an LVEF < 55%.

Left ventricular systolic and diastolic functions were also evaluated by using the PWD trace of the mitral valve and pulsed tissue Doppler at the basal septal and basal lateral segments of the mitral annulus.

Standard PWD parameters including the peak of early (E) and late (A) diastolic flow velocities were recorded at the tips of the mitral valve leaflets. Pulsed wave tissue Doppler was used to record the velocity profile of the mitral annulus from the apical 4-chambered view. The mean values of longitudinal myocardial velocities including peak early diastolic (e′), late diastolic (a′), and also peak systolic (s′) velocity were measured from the basal septal and basal lateral segments of the mitral annulus.

Then, we calculated the ratio of the peak diastolic flow velocity of mitral inflow (E) to peak e’ velocity (E/e′ ratio) as the best non-invasive marker of the LV filling pressure. According to the average of E/e′ ratio in the lateral and septal wall of the mitral valve, patients were categorized into three groups as follows: patients with E/e′ ≥ 14 (diastolic dysfunction and elevated filling pressure), patients with 8 < E/e‘ < 14 (suspected diastolic dysfunction), and those with E/e′ ≤ 8 (normal diastolic function) (25).

**FIGURE 1:**
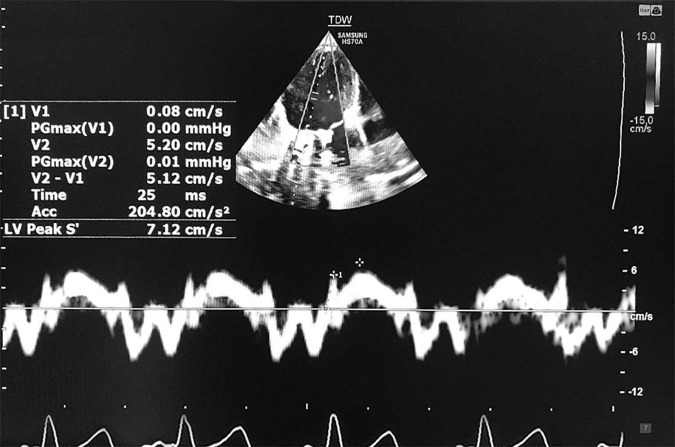
Measurement of tissue Doppler-derived IVA and s’ velocity. IVA, myocardial acceleration during isovolumic contraction; s′, systolic longitudinal myocardial velocity.

The LV-IVA, as an index of the contractile state of the myocardium, was also measured by TDI at the basal lateral side of the mitral annulus. It was defined as the first positive deflection in the tissue Doppler profile at the end of the a′ wave. A line was drawn from the onset of the wave to its peak, and then a slop was measured (Figure 1; 26).

**FIGURE 2:**
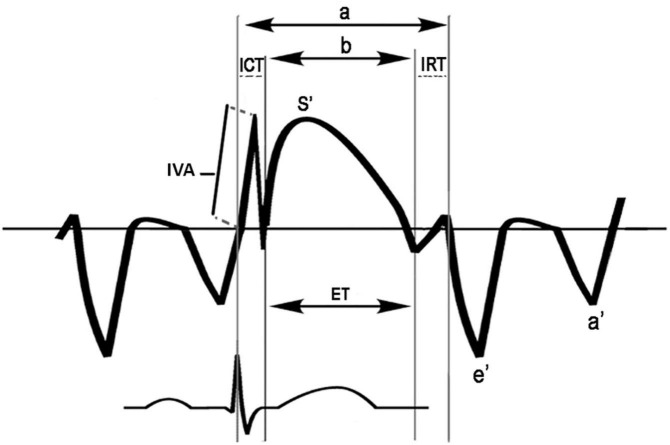
Scheme for measurement of time intervals used to calculate the tissue Doppler-derived myocardial performance index (TDI-MPI) and IVA: The MPI index was calculated as (a-b)/b; *a*, time from cessation of the a’ wave to the onset of the e’ wave; *b*, the duration of the s’ wave; ET, ejection time; ICT, tissue Doppler-derived isovolumic contraction time; IRT, tissue Doppler-derived isovolumic relaxation time; IVA, myocardial acceleration during isovolumic contraction. The IVA was defined as the first positive deflection in the tissue Doppler signal at the end of the a’ wave. A line was drawn from the IVA onset to its peak, and a slope was measured.

Moreover, the myocardial performance index (MPI), a marker of global ventricular function, was also determined by both PWD and TDI. To obtain PWD-MPI of the left ventricle, Doppler time intervals were recorded as proposed by Tei et al. TDI-MPI was calculated from the tissue Doppler spectra of the septal mitral annulus (Figure 2; 27–29).

### Statistical Analysis

Continuous data were checked for normality using the Kolmogorov-Smirnov test and expressed as mean ± SD or median as appropriate. Mean values were compared with each other in the study groups using the Student’s *t*-test or Mann–Whitney *U*-test, based on whether the variables followed a normal distribution or not. Pearson and linear regression analyses were utilized to evaluate potential relationships between variables. The appropriate cutoff values for variables were identified based on previous reports or using receiver-operator characteristic (ROC) curves. Then, a *t*-test was used to compare continuous variables (LVMI, MPI, s’velocity, etc.) between two categorical groups based on the identified cutoff values. The prediction of the global LV function was carried out through an analysis of the ROC curves for MPI. To assess intra-observer variability, the observer performed repeated measurements of all echocardiographic variables in 8 patients. Then, intraclass correlation coefficients were calculated using SPSS statistical package version 26 (SPSS Inc., Chicago, IL, United States). A *p*-value less than 0.05 was considered significant.

## Results

The demographic data and hematological profile of the study population are summarized in Table 1.

**TABLE 1:** Demographic and clinical characteristics of patients and control subjects*.

Variables	Patients *N* = 28	Control *N* = 35	*P*-value
Age	13.6 ± 5.7	14 ± 2.1	NS
Gender, Male/Female	12/16	18/17	
Weight (kg)	35.9 ± 13.1	37 ± 11	NS
Height (m)	1.41 ± 0.6	1.49 ± 0.7	NS
BSA (m^2^)	1.1 ± 0.29	1.2 ± 0.01	NS
HR (beat/min)	83 ± 9	80 ± 8	NS
SBP (mmHg)	110 ± 12	104 ± 10	NS
DBP (mmHg)	60 ± 5.6	61 ± 6.3	NS
Ferritin, (ng/mL)	488 ± 459		
Hb (g/dL)	9.3 ± 1		
Splenectomy	11		

*Data are presented as Number, (%) or Mean ± SD. NS, not significant; BSA, body surface area; HR, heart rate; SBP, systolic blood pressure; DBP, diastolic blood pressure.

History of the previous transfusion was observed in 11 patients (once in seven patients and three times in four patients). All of the subjects were normotensive. The heart rate in TI cases was not significantly different from that of the control group, and the body surface area did not differ between the two groups as well. Splenectomy was performed in 11 patients (39.2%). None of the patients revealed evidence of cardiac iron overload (mean cardiac T2* values = 33.2 ± 10.3 ms).

Ventricular diameters, LVMI, stroke volume, and cardiac index were all significantly greater in patients (Table 2). LVH was found in 13 (46.4%) patients. LVMI was correlated positively with the LV end-systolic dimension (*r* = 0.61, *p* < 0.05). However, no association was found between LVMI and age and also between the mean hemoglobin level and mean serum ferritin concentration.

**TABLE 2:** Geometry, cardiac volume, and cardiac index in patients and control subjects*.

Variables*	TI (*n* = 28)	Control (*n* = 35)	*P-*values
LVEF, %	62 ± 5.4	64 ± 3.9	NS
LV MI, gr/m^2^.^7^	50.13 ± 11.9	33 ± 9	<0.01
LVEDVI, ml/m^2^	79.6 ± 24.7	63 ± 12	<0.05
LVESVI, ml/m^2^	24.9 ± 10.2	20 ± 6.1	<0.05
LVDDI, cm/m^2^	3.69 ± 0.58	3.03 ± 0.12	<0.05
LVSDI, cm/m^2^	2.3 ± 0.47	2.1 ± 0.31	<0.05
SVI, ml/m^2^	53.4 ± 15.2	41 ± 6.7	<0.01
CI, ml/min/m^2^	4,211 ± 1,227	2745 ± 549	<0.01

*Values given as mean ± SD. LVMI, left ventricular mass index; LVEDVI, left ventricular end diastolic volume index; LVESVI, left ventricular end systolic volume index; LVDDI, left ventricular end diastolic diameter index; LVSDI, left ventricular end systolic diameter index; SVI, stroke volume index; CI, cardiac index.

The LVEF was within the normal limits for all patients (62 ± 5.4%). Regarding the LV longitudinal systolic function, the myocardial systolic velocity (s′) was significantly different between the patients and control group (8.1 ± 1.4 vs. 9.1 ± 0.7 cm/s, *p* < 0.05), but myocardial diastolic velocities did not differ between patients with TI and control subjects (Table 3).

**TABLE 3:** Left ventricular (LV) systolic and diastolic function using pulsed Doppler (PWD) and tissue Doppler imaging (TDI) echocardiography*.

Variables	Patients (*N* = 28) Mean ± SD	Controls (*N* = 35) Mean ± SD	*P*-value
Peak E, cm/s	131 ± 15	108 ± 9	<0.05
Peak A, cm/s	77 ± 19	68 ± 25	NS
PWD-MPI	0.37 ± 0.08	0.32 ± 0.03	<0.03
TDI-MPI	0.40 ± 0.05	0.32 ± 0.02	<0.001
e′, cm/s	14.4 ± 0.8	15.1 ± 1	NS
a′, cm/s	7.1 ± 2.9	7.9 ± 3.7	NS
s′, cm/s	8.1 ± 1.4	9.1 ± 0.7	<0.05
E/e′ ratio, average	9 ± 1	6 ± 1	<0.05
IVA, septal, m/s^2^	2.18 ± 0.21	2.54 ± 0.53	NS

*Data are presented as mean ± SD. NS, not significant; E, early diastolic pulsed-wave Doppler velocity; A, late diastolic pulsed Doppler velocity; PWD-MPI, pulsed Doppler myocardial performance index; TDI-MPI, tissue Doppler-derived myocardial performance index; e′, peak early mitral annular velocity during diastole; a′, peak late mitral annular velocity during diastole; s′, peak systolic mitral annular velocity; IVA, myocardial acceleration during isovolumic contraction.

Moreover, an inverse relationship was observed between the s′ wave velocity and LVMI (*r* = –0.46, *p* < 0.05). According to data from the literature, we considered an s′ wave value less than 7.9 cm/s as a cut point for impaired LV longitudinal contractility. Accordingly, a reduced peak systolic velocity was observed in nine patients (32.1%). Mean age and LVMI were significantly higher in patients with s′ < 7.9 cm/s in comparison with those with s′ > 8 cm/s (14 ± 5.9 vs. 12 ± 4.2 years, *p* < 0.05 and 54.7 ± 11 vs. 48.3 ± 9 g/h^2.7^, *P* < 0.05, respectively). Our finding is in favor of the essential effect of progressive LV remodeling on impaired longitudinal myocardial contractility. Association was observed neither between the s′ velocity and mean hemoglobin, nor between the serum ferritin level and s′ velocity. When we assessed the LV contractility state by the measurement of IVA, no significantly different values were found between the patients and control group (2.18 ± 0.21 vs. 2.54 ± 0.53 m/s^2^, *p* = 0.68). However, the IVA value was observed to have a negative relationship with the existence of LVH in patients (*r* = –0.69, *P* < 0.05).

Evaluation of the LV diastolic function at mitral inflow displayed an increased peak with early and late filling velocities that were compatible with the high cardiac output state; however, only the peak E-value was significantly higher in patients compared to controls (131 ± 15 vs. 108 ± 9 cm/s, *p* < 0.05). Furthermore, we found a higher E/e′ ratio in patients with TI in comparison with control subjects, which is in favor of the abnormal LV diastolic function (Table 3). None of the patients had high filling pressure (E/e′ ratio > 14), while normal LV filling pressure (E/e′ ratio < 8) was only observed in seven patients (25%). Statistical analysis performed to determine associated echocardiographic parameters showed that the LV end-systolic and end-diastolic diameters have a significant relationship with the E/e′ ratio (*r* = 0.58, *p* < 0.05, *r* = 0.64, *p* < 0.05, respectively). The peak systolic myocardial values (s′) were lower in the patients with E/e′ > 8 in comparison with the patients with a normal E/e′ ratio, but the difference was not significant (7.8 ± 0.1 vs. 8.2 ± 0.9 cm/s, *p* < 0.05). No significant correlation was found between the serum ferritin level and E/e′ ratio either.

**FIGURE 3:**
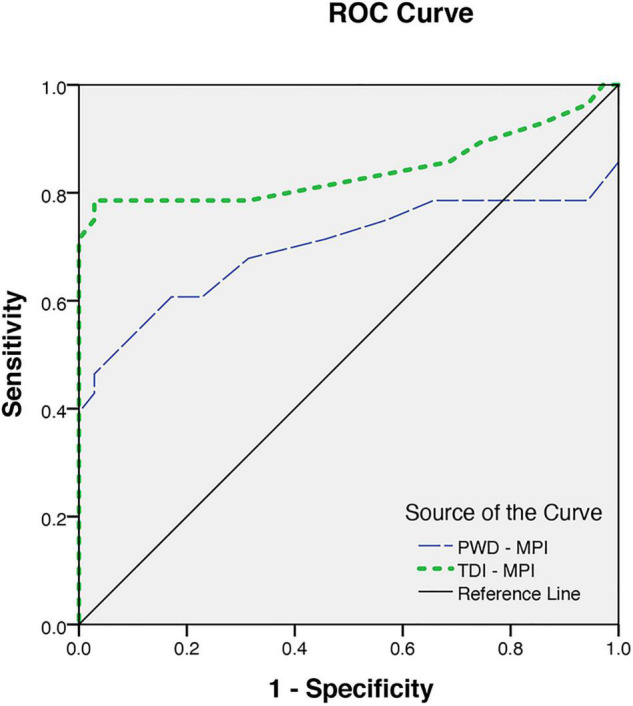
The receiver operating characteristics for myocardial performance index (MPI) obtained from pulsed Doppler (PWD-MPI) and tissue Doppler-derived MPI (TDI-MPI).

The MPI, as an indicator of the global LV function obtained by the tissue Doppler method, was significantly higher in patients than in the control group (0.40 ± 0.05 vs. 0.32 ± 0.02, *p* < 0.001). Considering the ROC curve analysis, the TDI -MPI produced an area under the curve of 0.84 to discriminate the patients with and without latent LV dysfunction. Choosing a TDI-MPI > 0.33 as a cutoff value, the LV dysfunction was found with a sensitivity of 78% and a specificity of 80%. The obtained values for PWD-MPI were also higher than the values in normal subjects (0.37 ± 0.08 vs. 0.32 ± 0.03, *p* < 0.03). The PWD-MPI cutoff value of >0.35 indicated subclinical LV dysfunction with a sensitivity and specificity of 64 and 91%, respectively (Figure 3). However, the MPI obtained by tissue Doppler echocardiography was identified to have a positive correlation with the presence of both LVH (*r* = 0.64, *P* < 0.05) and increased the LV end-systolic volume diameter (*r* = 0.4, *p* < 0.05). These findings highlight the essential role of chronic volume overload in the pathogenesis of the LV dysfunction.

Intraclass correlation coefficients were good for IVA, E/e, E velocity, s’ velocity, and TDI-MPI (0.77–0.88), and moderate for e’ velocity (0.72).

## Discussion

The left ventricular function has not been widely investigated in children or adolescence with TI, a disease that has been usually characterized by late-onset pulmonary hypertension followed by right heart failure. In this study, using more sensitive echocardiographic methods, abnormal LV geometry and slight impairment in the LV function were identified in young patients with TI who had no evidence of cardiac iron overload.

Individuals with TI often have a high cardiac output state and increased LV dimensions secondary to chronic hemolytic anemia (13, 30). Different patterns of LV remodeling have been reported in patients with chronic volume or pressure overload conditions (8, 31, 32). The LVH has been documented as an important risk factor for cardiovascular disease morbidity and mortality (31, 33–35). Moreover, a recent study by Hieda et al. (36) demonstrated that the LVH and its myocardial stiffness effect have a predisposing role in the development of progressive heart failure. Our findings regarding increased LV mass and its correlation with greater values of TDI-MPI and also lower s′ wave values were in concordance with previous reports and highlight the leading role of the LVH in producing subtle ventricular impairment (36, 37).

The obtained high E wave velocities were associated with increased LV end-systolic volume (*r* = 0.56) and with greater LV mass as well (*r* = 0.54) that were compatible with a high cardiac output state secondary to chronic anemia. These findings are in concordance with previously reported data on more increased LV volumes and mass in patients with TI than in those with the thalassemia major. These studies also emphasized the essential role of the chronic high cardiac output state in the pathogenesis of LV remodeling (9, 11, 38). Long-term effects of chronic hemolytic anemia on the cardiovascular system augment gradually with age; thus, progressive LV remodeling remains a mandatory mechanism to maintain the permanent high cardiac output state in thalassemia patients. As a result, patients with TI who have chronic and mild anemia can be at risk for progressive cardiovascular complications (5, 39).

However, most of the previous data reported in the literature are somewhat conflicting because most of the conventional echocardiographic parameters, mainly LVEF, are strongly influenced by the high cardiac output state. In fact, Westwood et al. (40) showed that thalassemia patients with a normal iron load have greater values for the LV volumes and also higher LVEF compared to controls. These different values have the potential to lead to delayed diagnosis of thalassemia cardiomyopathy in this group of patients. Accordingly, recent studies focused on new and reliable echocardiographic indices for early diagnosis of subtle myocardial dysfunction (17, 41–43). More recent studies suggested that early diagnosis of ventricular impairment using MPI could improve cardiovascular prognosis (44, 45). Similarly, we identified increased TDI_MPI values in patients with TI who had the preserved systolic function by using conventional echocardiographic parameters. Also, we observed a correlation between the values of PWD-MPI and the increased LV systolic volume index (*r* = 0.4, *p* < 0.01). Our observation was in line with previous studies and supported the primary role of the high cardiac output state and its compensatory reactions for developing cardiac complications in patients with TI without iron overload (8, 9, 40). However, certain previous studies demonstrated that PWD-MPI was influenced by the LV preload and heart rate variability, whereas the TDI-derived MPI was a promising index for the evaluation of the ventricular function with less load and heart rate dependency (29, 46, 47). As presented in our study, the TDI-MPI has a significant sensitivity and specificity for predicting global LV dysfunction. Our finding was in line with previous studies on the sensitivity of TDI-MPI for the LV function assessment in children with congenital heart disease and chronic kidney disease (47, 48).

Although the utility of the TDI-MPI for detecting the asymptomatic myocardial dysfunction was determined in patients with the thalassemia major, no data regarding the measurement of the TDI-MPI in children with TI are available in the literature (49). Thus, no comparison is possible regarding patients with TI because of different pathophysiologic mechanisms for developing the LV abnormalities in this group. However, our finding regarding the increased value of the TDI-MPI in patients with a greater LV mass index is in line with the report of Ucar and colleagues on thalassemia syndrome (49). Having excluded the cardiac iron overload using CMR T2*, we are inclined to attribute these abnormal values of global ventricular function to cardiac maladaptive response secondary to the high cardiac output state that occurred early in young patients with the TI.

Previous studies have shown the essential role of longitudinal fiber shortening in global ventricular contraction. Also, earlier impairment of longitudinal function in comparison with circumferential fiber shortening of the LV myocardium has been found in a variety of cardiac conditions. Therefore, assessment of longitudinal myocardial systolic velocity (s′) by the TDI, which is relatively independent of volume load, may give early and reliable information to identify subclinical myocardial dysfunction in comparison with conventional methods (50–52). In agreement with previous reports, we found significantly reduced s′ wave velocity in patients with TI with normal LVEF (17, 52). Furthermore, the association between the s′ wave velocity and the LVMI was suggestive of the basic role of abnormal LV geometry in the development of longitudinal systolic dysfunction.

Although the TDI-derived systolic myocardial velocity is accepted as a reliable parameter for detecting subclinical LV dysfunction, the measurement of the IVA may provide further information on myocardial contractility in patients with abnormal geometry (18, 53). The measurement of the IVA that is relatively not load-dependent offers important benefits to estimate the LV function in the thalassemia patients due to the confounding effect of their anemic state on echocardiographic ejection phase indices. Recently, Cheung et al. (54) demonstrated the relationship between the LV-IVA and myocardial iron load in patients with the TM, suggesting the impairment of the LV contractile reserve and the potential pathogenic role of myocardial iron.

Despite the increasing use of the IVA as an index of the myocardial systolic function, the IVA values which may predict LV systolic dysfunction in children have not yet been fully established (55, 56). Although our study did not find any significant difference in the IVA values between patients and controls, its values for the patients with the LVH were significantly lower than those of the patients with a normal LV mass index (2.09 ± 0.56 vs. 2.26 ± 0.23 m/s^2^, *p* < 0.05), suggesting the primary role of the LV remodeling on impaired myocardial longitudinal contractility. However, no correlation was found between the IVA and LVEF in patients with and without the LVH. Our findings regarding the diagnostic value of the IVA in detecting subtle myocardial contraction abnormalities were in concordance with findings of other researchers in patients with congenital heart disease or hypertensive cardiomyopathy (57, 58). However, more investigation is needed for confirming its exact value for detecting subclinical ventricular dysfunction in children with the TI who suffer from multiple confounding factors or adaptive mechanisms that interfere with the cardiovascular function.

Previous studies describing the LV diastolic function have shown more conflicting data in patients with TI. Some studies demonstrated that both the LV volume loading and high cardiac output level influenced the echocardiographic measurements of the LV diastolic function, mimicking a restrictive pattern of mitral inflow when assessed by conventional methods (9, 38). Recently, the TDI measurement of mitral annulus diastolic velocities has been shown as a more accurate method for evaluating the diastolic function due to less dependency on the HR or preload (25, 59). According to the data reported previously, the E/e′ ratio has the best correlation with invasively obtained LV filling pressure (60, 61). Likewise, recent studies revealed that it can be used for the identification of latent diastolic dysfunction in thalassemia patients (62, 63). In addition, its relationship with the myocardial iron loading condition was demonstrated in the study by Silvilairat et al. (64). Our finding regarding the significantly elevated E/e′ ratio, which is in agreement with the finding of other researchers, has suggested an early development of diastolic function impairment in patients with TI (Table 3; 17, 38, 63). Although, the average E/e ratio > 8 was found in 75% of the patients, none of them had high filling pressure (E/e ratio > 14). Furthermore, a significant relationship was observed between the E/e′ ratio and both of the LV systolic and diastolic diameters respectively, indicating somewhat diastolic abnormalities secondary to maladaptive LV compensatory mechanisms. However, we found no significant difference in the LV mass by comparing the patients with or without E/e′ ratio > 8. This finding could be explained by the best relationship of this Doppler parameter with the early phase of the LV filling while the LV hypertrophy affects the ventricular stiffness of the myocardium, which is known to affect the later phase of the diastolic function. The patients with the increased E/e′ ratio showed a mild abnormality of systolic myocardial velocity in comparison with patients with the normal diastolic function (E/e < 8). Our finding confirms the adverse effect of the LV remodeling on both systolic and diastolic myocardial functions in young patients with TI without heart failure. Our observation is in concordance with the data of Marci et al. (17) who found abnormal myocardial tissue velocities as a marker for developing adverse cardiac events. However, it has been shown that wide variability in the LV filling pressure may be observed in those with E/e′ of 8–14, and in this situation, the other information must be applied. Previous research studies also point out the importance of considering additional echocardiographic parameters or biochemical markers for a more accurate evaluation of filling pressures in the group with the intermediate values of E/e.

### Limitations

The relatively small population of the study subjects is a limitation of our research that precludes a more perfect evaluation of echocardiographic indices of the LV function; however, the population size satisfies the minimum requirements of the statistical tests conducted.

Although the E/e′ ratio was shown as the sensitive Doppler measurement to estimate the LV filling pressures in patients with the preserved systolic function, the presence of a single index, which does not fall within the normal range, does not necessarily indicate the abnormal diastolic function. Consequently, the lack of further characterization of the group with the intermediate E/e′, due to small population of patients, is the main limitation of this study. In contrast, because of the lack of diastolic function parameters validated for younger patients, we have to rely on recent guidelines derived from adult studies. Moreover, previous studies have shown a wide range of normal values in children which adversely affect the interpretation of the diastolic function.

The use of the apical echocardiographic views to assess the mitral annulus velocities is an attempt to focus on the long-axis motion of the left ventricular myofibrils. However, the motion of the mitral annulus is not entirely due to longitudinal contraction but rather is the sum of longitudinal, spiral, and circumferential contraction. The effects of each of these may vary from a patient with normal geometry to those with the LV remodeling. Although other studies have demonstrated the impact of the IVA for assessing ventricles with abnormal geometry, the greatest limitation of the IVA is reproducibility.

## Conclusion

We presented substantial LV remodeling, and subtle systolic and diastolic abnormalities in asymptomatic young patients with TI with normal cardiac iron concentration. Our findings suggest that the LV remodeling, which is a primary and basic adaptation response to high cardiac output state, might be the first stage in developing the LV failure.

Both myocardial systolic velocity and the MPI obtained by the TDI appear to be sensitive parameters for detecting asymptomatic thalassemic myocardial dysfunction. The increased value of the E/e′ ratio in the majority of the patients may be considered an early indicator of latent diastolic impairment. The prognostic significance of these findings for the identification of patients at risk of progressive heart failure and also the diagnostic impact of the above-mentioned parameters to modify the routine approach for follow-up of these patients are yet to be confirmed by further studies.

## Data Availability Statement

The raw data supporting the conclusions of this article will be made available by the authors, without undue reservation.

## Ethics Statement

The ethics committee approved the study of the Iran University of Medical Sciences with the code number: IR.IUMS.REC 90-02-30-13156, and all patients signed an informed written consent form prior to entering the study. Written informed consent to participate in this study was provided by the participants’ legal guardian/next of kin.

## Author Contributions

RI and KA contributed to the conception and design of the study. RI and FS wrote the sections of the manuscript. RI performed the statistical analysis. KA and MR collected the patient’s information. All authors contributed to manuscript revision, read, and approved the submitted version.

## Conflict of Interest

The authors declare that the research was conducted in the absence of any commercial or financial relationships that could be construed as a potential conflict of interest.

## Publisher’s Note

All claims expressed in this article are solely those of the authors and do not necessarily represent those of their affiliated organizations, or those of the publisher, the editors and the reviewers. Any product that may be evaluated in this article, or claim that may be made by its manufacturer, is not guaranteed or endorsed by the publisher.
